# Gateway to Care campaign: a public health initiative to reduce the burden of hepatitis B in Haimen City, China

**DOI:** 10.1186/1471-2458-14-754

**Published:** 2014-07-27

**Authors:** Gang Chen, Joan M Block, Alison A Evans, Peixin Huang, Chari Cohen

**Affiliations:** Hepatitis B Foundation, 3805 Old Easton Road, Doylestown, PA 18902 USA; Drexel University, Philadelphia, PA USA; Haimen City Center for Disease Control and Prevention (HCCDC), Haimen City, China

**Keywords:** Chronic hepatitis B infection, Hepatocellular carcinoma, Perinatal hepatitis B, Prevention, Health education campaign, Hepatitis B screening, Hepatitis B vaccination, Haimen City, China

## Abstract

**Background:**

An estimated one million people worldwide die each year from complications of chronic hepatitis B infection (CHB), including liver cancer. A disproportionate number of infections and deaths occur in China. The incidence and mortality of liver cancer in Haimen City is among the highest in China, and in the world. A multi-year citywide campaign was aimed at eliminating hepatitis B virus (HBV) infection and significantly reducing the number of liver cancer deaths due to CHB in Haimen City, China.

**Methods:**

Strategies included a public health information campaign targeting the 1.03 million city residents; specialized health education for leaders and providers to increase adoption of evidence-based HBV management protocols; establishment of health care infrastructure and management systems; and increased prevention and care delivery to key subpopulations (especially pregnant women).

**Results:**

The project developed and deployed broad-reaching public awareness and health education tools and modules to 280,000 households and at community-based events. More than 90% of targeted healthcare providers and 80% of the community leaders/government officials attended educational seminars during the project period (1,441 health care providers; 1,883 local government officials). A centralized registration and management system for pregnant women was developed and instituted, 100% of pregnant women were enrolled (5,407 women over one year), and all infants born to HBV-infected mothers received one dose of HBIG and the first dose of HBV vaccine by 24 hours of birth.

**Conclusions:**

Lessons from the implementation phase of the project include the importance of: gaining early and ongoing support from the local government and health bureau for success in reaching the targeted populations; and having project management by a local, experienced, and trusted health expert to navigate implementation and relationships, and help develop culturally and linguistically appropriate materials.

**Electronic supplementary material:**

The online version of this article (doi:10.1186/1471-2458-14-754) contains supplementary material, which is available to authorized users.

## Background

### The burden of chronic hepatitis B infection in china

Worldwide, an estimated two billion people have been infected with the hepatitis B virus (HBV) and, of these, nearly 400 million remain chronically infected [1, 2]. About one-third of those with chronic hepatitis B (CHB) infection live in China, where it is estimated that around 100 million people (about 10% of the Chinese population) are chronically infected, and around 500,000 people die from CHB-related complications, including primary liver cancer (hepatocellular carcinoma, HCC) and cirrhosis, each year [3–6]. China has the highest age-adjusted incidence (new cases) of HCC due to CHB, and about 55% of liver cancer deaths worldwide occur in China [7]. Most of these deaths are men in their prime, between the ages of 30 and 65 years. Up to 9.5% of Chinese women of childbearing age have CHB [8]. Newborns infected perinatally have a more than 90% risk of developing CHB infection, and thus have an increased risk of premature death from end-stage liver disease or liver cancer [9, 10]. Of babies born to HBV-infected mothers, 5% to 10% become chronically infected despite receiving immunoprophylaxis with Hepatitis B immune globulin (HBIG) and hepatitis B vaccine [11].

There are significant unmet needs for millions of individuals living with CHB, including a lack of screening and diagnosis, and limited access to appropriate medical care and treatment. The Hepatitis B Foundation (HBF) believes that the burden of CHB can be reduced, and lives saved, through improved public awareness, health education, and screening. To this end, HBF launched the “Gateway to Care Campaign: Haimen City Project” in August 2010, in collaboration with the Haimen City Center for Disease Control and Prevention (HCCDC). This three-year project consisted of a targeted citywide public health information campaign; specialized health education and training for providers; establishment of health care infrastructure and management systems; and increased screening, vaccination, treatment, and care management service delivery to key subpopulations (especially pregnant women and their infants and families).

### The Haimen City cohort

Haimen City is located in Jiangsu Province, approximately 60 miles northwest of Shanghai, with a total population of 1.03 million. HCC has been the leading cause of cancer death in this population since 1970 (when death registration began), and the incidence and mortality of HCC in Haimen City are among the highest in China, and in the world (Haimen City CDC Vital Statistics, unpublished data). A landmark study of this population published in 2006 clearly established a direct link between hepatitis B viral load and subsequent risk of liver cancer in chronically infected individuals [12]. This population-based prospective cohort study, begun in 1992, enrolled more than 90,000 Haimen City residents aged 25 to 64 years old. Blood tests for the presence of the hepatitis B surface antigen (HBsAg) indicated a 13.7% HBV infection rate of the cohort. Intensive follow-up, including annual collection and validation of vital statistics of each cohort member, has been conducted since 1994. Between 1994 and 2007, there were 7,468 deaths in the cohort, of which 1,717 (23%) were due to HCC.

### Critical gaps in knowledge and practice

Previously published findings from this cohort have contributed to global knowledge regarding the outcome of chronic HBV infection. Although evidence-based HBV prevention, treatment, and management protocols have been published [13–17], evidenced-based guidelines have not been implemented in Haimen City. Huge gaps in knowledge and practice persist in the general population, among patients, and at every level of health care providers in Haimen City. City public health officials estimate there are approximately 80,000 people between the ages 25 to 64 years in Haimen City with CHB. Thousands will likely die from HCC and other chronic liver diseases within the next ten years if no appropriate actions are taken towards the prevention and control of HBV in this population.

## Methods

### Project location and collaborators

Over the past two decades, Haimen City residents have had high rates of participation in epidemiologic studies of the impact of HBV on their population [12, 18, 19]. However, Haimen City (and much of China) still suffers the stigma, loss of productivity, and death resulting from hepatitis B. HBF saw a critical need to address the significant burden of CHB through community-based education and the creation of sustainable public health services that could serve as a model for implementation in other areas of China, and other high HBV-endemic areas around the world. Excellent working relationships between HBF and the HCCDC, the Haimen City Health Bureau, Haimen City Center for the Health of Women and Children (HCHWC), and the Haimen City Municipal Government, made Haimen City an appropriate site for this demonstration project. The HCCDC has an excellent track record in public health awareness programming; the recruitment and retention of study subjects; the collection of high quality data and biological samples; and the training of doctors and other public health professionals whose participation is needed to implement the project activities.

Specific benefits of conducting the campaign in Haimen City included: immediate start-up of the project due to existing infrastructure and relationships; timely government approval of the program; comprehensive, well-organized health care infrastructure; cooperation of healthcare officials; local experience in working with U.S. collaborators; accurate data collection and credible results; and the potential for the transferability of the program to other small city and rural populations.

### Target populations

The public awareness component of the campaign was designed to reach as many city residents as possible (total 1.03 million population, 280,000 households). A more targeted educational outreach was directed toward city and township government officials; school nurses; obstetricians and nurse assistants; public health officials and physicians from 23 townships; village physicians from 239 villages; and community leaders from 239 villages. In addition, because transmission of HBV from mother to child during the perinatal period remains a serious medical problem in China, the campaign sought to reach all pregnant women over a one-year period; HBV-infected pregnant women and their newborns over a one-year period; and spouses and first-degree relatives of HBV-infected pregnant women.

### Program goals and activities

The Gateway to Care Campaign has three primary goals focused on public awareness, provider and leader education, and elimination of perinatal transmission of HBV (Table 1). Multi-platform educational campaigns for the general public were developed and deployed, highlighting the importance of being tested for HBV (see Additional file 1). Specialized educational seminars were developed for government officials, community leaders, and healthcare providers to increase their knowledge about HBV infection and HCC, and foster appropriate testing and referral to care. Finally, the project sought to increase uptake by Chinese obstetrical and perinatal care providers of improved, evidence-based methods to prevent and manage perinatal HBV infection (see Table 1 for details on strategies and targets). The educational materials, training modules, survey questionnaires, and comprehensive, multi-modal outcome evaluation plan were developed together with the local project manager who is on staff at the HCCDC (author PH).

**Table 1:** **Gateway to care campaign: Haimen city project goals, srategies and targets**

**Goals**		
**1) Raise public awareness**	**2) Educate providers and leaders**	**3) Prevent perinatal transmission**
Implement a citywide public health information campaign to increase understanding and acceptance of current knowledge about hepatitis and liver cancer among the general public.	Educate government officials, community leaders, and healthcare providers to increase their knowledge about hepatitis B and liver cancer.	Initiate evidence-based practices for pregnant women to prevent perinatal transmission of hepatitis B and to ensure appropriate care and referral of chronically infected women.
**Strategies**		
• Initiate a culturally competent public awareness campaign to inform the public about hepatitis B and the importance of prevention, screening and management of this serious liver infection in partnership with the Haimen City Health Bureau and HCCDC.	• Educate city and township government officials to gain their support of public health programs. Work with Haimen City government to present seminars to city and township officials who can play an important role in supporting critical public health initiatives, and in reducing social stigma and discrimination.	• Evaluate current practices in perinatal care for HBV-infected and non-infected pregnant women. Work with HCCHWC to retrospectively review HBV testing, follow-up, and antepartum treatment of 1,000 pregnant women, and passive-active immunization of neonates after birth ( including completion rates of the three shot series)
• Disseminate information in partnership with and through the comprehensive HCCDC network of city hospitals, city and township health centers, and village doctors.	• Educate community leaders to gain their support of public health programs. Work with HCCDC to present seminars to the village head, party secretary, accountant, and women’s representatives from 239 villages in Haimen City who will, in turn, distribute educational materials to residents of their villages.	• Establish a centralized registration, and management system for all pregnant women in the city to ensure appropriate screening and care. Work with HCCHWC to create an interactive system to register and estimated 6,000 pregnant women in the city, and input data from HBV-positive women and their newborns.
• Create simple and appealing educational literature, posters and interactive materials (e.g., calendars, posters, playing cards, paper cups). Coordinate media outreach through print, televisions, and broadcast outlets to include news articles, personal stories, and community events. Key message: hepatitis B can lead to liver cancer, but it can be prevented with a safe vaccine and managed with effective treatments.	• Educate township-level public health officials and physicians to emphasize the importance of testing and referral to care. Develop health education curricula and work with HCCDC to present seminars to public health officials and physicians at township-level health care centers and hospitals.	• Conduct a prospective study to evaluate failures of perinatal immunoprophylaxis (HBIG and HBV vaccine). HBV DNA quantitation will be used to determine if there is any correlation between high maternal viral load and perinatal prophylactic failure.
• Develop targeted educational materials for individuals with CHB to increase their knowledge about hepatitis B and liver cancer and encourage them to seek and maintain regular care.	• Educate community-based physicians about importance of prevention, screening, management, and referral to liver specialists. Work with HCCDC to present seminars to village doctors from 239 villages.	• Develop a standard protocol to educate and test spouses and relatives of all pregnant women. Education will be provided to family members to increase their knowledge about HBV abd reduce the social stigma of hepatitis B.
	• Educate school nurses about HBV transmission, vaccination of susceptible children, and preventing discrimination against infected children. Work with the Haimen City Educational Bureau to present seminars to school nurses at primary and secondary schools in the city.	
	• Educate obstetricians and midwives about prevention of perinatal HBV transmission. Work with HCCHWC to present seminars to obstetrical providers in the city. Develop evidence-based screening, vaccination, and HBV management guidelines for pregnant women.	
**Targets**		
Disease awareness information distribution to healthcare centers and administrative buildings in all 239 villages, as well as hospitals, and health service centers in all 23 townships.	At least 90% of government officials and community leaders and 100% of village doctors, obstetricians, and school nurses will have attended at least one formal education session.	100% of newly registered pregnant women screened for HBV and infected women followed under evidence-based protocols over a 12-month period. 100% of newborns to receive HBV vaccine and HBIG with 12 hours of birth. 100% of infants born to infected mothers to receive HBV vaccine and HBIG within 12 hours of birth.

CHB, chronic hepatitis B infection; HBIG, hepatitis B immune globulin; HBsAg, hepatitis B surface antigen; HBV, hepatitis B virus; HCCDC, Haimen City Center for Disease Control; HCCHWC, Haimen CIty Center for the Health of Women and Children.

## Results and discussion

**Figure 1:**
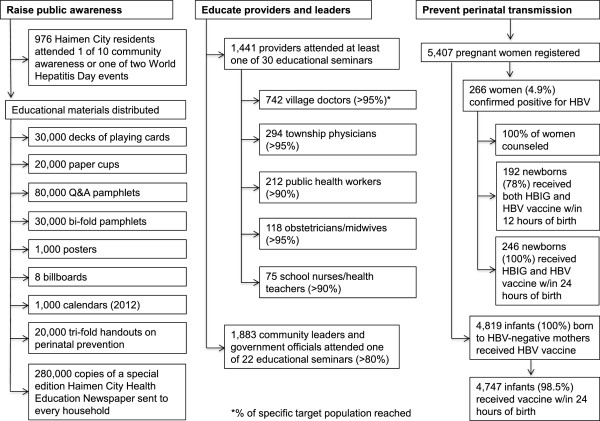
**Overview of outreach activities.** Numbers and proportion of Haimen city residents, city leaders, health care providers, and pregnant women reached during the implementation phase of the project.

The implementation phase of the Haimen City project concluded in July 2013. Figure 1 summarizes the outreach activities and results for the three goals of public awareness, provider and leader education, and elimination of perinatal transmission. Overall, more than 90% of targeted healthcare providers and 80% of the community leaders/government officials attended educational seminars during the project period (1,441 health care providers; 1,883 local government officials; see Figure 1 for details).

A centralized registration and management system for pregnant women was developed and instituted, and 5,407 pregnant women were registered and screened for hepatitis B between July 1, 2011 and June 30, 2012 (see Figure 1). A total of 266 women (4.9%) tested positive for HBsAg, and all 246 infants (100%) born to these HBV-infected mothers received one dose of HBIG and the first dose of HBV vaccine by 24 hours of birth. For comparison, an associated retrospective survey found that, of 180 infants born to HBV-infected mothers in Haimen City in 2010, 178 (98.9%) had received the first dose of HBV vaccine after birth, but only 128 infants (71.1%) had received HBIG (Haimen City CDC unpublished data).

Hepatitis B-related knowledge change in health care providers, health officials, and community leaders who attended educational events will be evaluated using data from pre- and post-tests conducted before and immediately after educational events, and two years after the launch of the campaign (to determine sustainability of change). The long-term impact of the project overall (sustainability of knowledge improvements, behavior change, systemic effects of the program/policy changes in Haimen City) will be assessed via focus groups, interviews, and surveys conducted at 1 and 3 years post-project completion. Data collection from the prospective study to evaluate perinatal prophylaxis failure is ongoing, and will be analyzed according to the approved study protocol.

## Conclusions

To our knowledge, this citywide awareness campaign designed to reach all 1.03 million Haimen City residents and their healthcare providers is the first of its kind addressing hepatitis B in China. Through its Gateway to Care initiative, HBF hopes to increase public awareness and health care provider knowledge about HBV prevention, screening, and care; expand HBV screening of pregnant women and ensure post-exposure prophylaxis of newborns; and foster adoption of evidence-based HBV management protocols. The HBF and HCCDC will continue to monitor the effects of the program for the next ten years, tracking expected reductions in deaths from CHB and HCC in the population of Haimen City. It is expected that increasing public awareness about hepatitis B will also help to dispel myths, and decrease the stigma and discrimination associated with this chronic and highly prevalent disease. The research findings about HBV knowledge and practices will contribute to the development of effective educational strategies for target audiences. These findings will also inform policy changes toward the improvement of health care outcomes in individuals with CHB, and the elimination of new HBV infections in China and other areas of the world where HBV is endemic. Deployment of the tools developed for this project, including the educational materials and the database system for registration and management of pregnant women, can also be expanded to other regions where HBV is endemic.

Several practical elements of the development and implementation phases of this project were contributors to its success thus far. Involving key stakeholders, including the Haimen City municipal government, the health bureau, and prominent physicians, at the beginning of the project was critical for success in reaching out to the targeted population in a project of this scale. HBF has established and maintained communication with the Chinese Foundation for Hepatitis Prevention and Control, in partnership with Haimen City government and public health officials, to pave the way for policy changes based on current and forthcoming project data. Project management conducted at the local level by an experienced and trusted member of the local health care community was extremely helpful for navigating implementation in Haimen City and its villages. (In this case the Deputy Director at the HCCDC was recruited as the project manager.) The onsite project manager oversaw the health educators and case managers, assured that milestones were met in a timely fashion, oversaw data collection and evaluation, worked with media outlets to promote the campaign, and interacted with the city public health and government leaders. Also essential was the development of culturally and linguistically appropriate, relevant, and potentially effective educational materials, training modules, and survey questionnaires. The local project manager and other key local experts are important resources for this task.

### Ethics statement

This program was reviewed and approved by the Medical Ethics Review Committee of Haimen City. No identifying or individual level data were collected as part of the demonstration (education) component of the program, thus the ethics committee considered this component to be exempt from full review.

## Electronic supplementary material

Additional file 1: **Community awareness campaign logo.** Residents of Haimen City received free hepatitis B education at community events and also through printed media and mailings. At community awareness events held in 2011, three hepatologists from the Infectious Disease Department of Haimen City People’s Hospital and three project staff from the HCCDC answered questions and distributed educational give-away items, including brochures and playing cards. All materials were branded with the program logo, seen here, which includes the messages “Right Knowledge, Right Action” and “Stopping Liver Cancer Starts from Prevention and Treatment of Hepatitis B.” (DOCX 388 KB)

## Abbreviations

(CHB): Chronic hepatitis B infection
(HBV): Hepatitis B virus
(HBF): Hepatitis B Foundation
(HCCDC): Haimen City Center for Disease Control and Prevention
(HBsAg): Hepatitis B surface antigen
(HCHWC): Haimen City Center for the Health of Women and Children
(HCC): Hepatocellular carcinoma
(HBIG): Hepatitis B immune globulin.
