# Supplementary figures and images for: Gateway to Care campaign: a public health initiative to reduce the burden of hepatitis B in Haimen City, China
Source: BMC Public Health. 2014 Jul 27;14:754. doi: 10.1186/1471-2458-14-754 (PMC4124160; doi:10.1186/1471-2458-14-754)

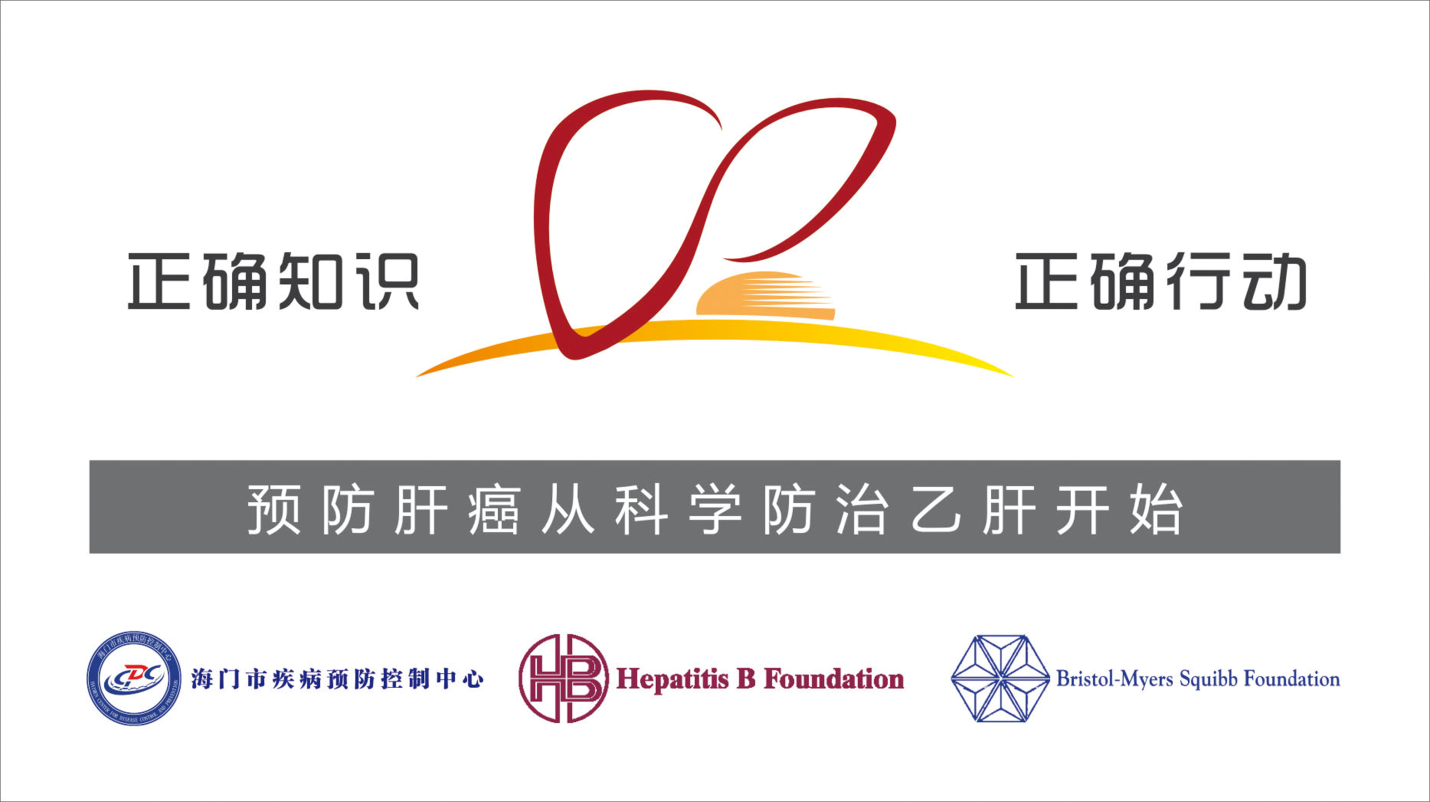

Supplement: Supplementary file 1 — Additional file 1: Community awareness campaign logo. Residents of Haimen City received free hepatitis B education at community events and also through printed media and mailings. At community awareness events held in 2011, three hepatologists from the Infectious Disease Department of Haimen City People’s Hospital and three project staff from the HCCDC answered questions and distributed educational give-away items, including brochures and playing cards. All materials were branded with the program logo, seen here, which includes the messages “Right Knowledge, Right Action” and “Stopping Liver Cancer Starts from Prevention and Treatment of Hepatitis B.” (DOCX 388 KB) [file 12889_2014_6892_MOESM1_ESM.docx]
